# Crystal structures of SARS-CoV-2 ADP-ribose phosphatase: from the apo form to ligand complexes

**DOI:** 10.1107/S2052252520009653

**Published:** 2020-07-17

**Authors:** Karolina Michalska, Youngchang Kim, Robert Jedrzejczak, Natalia I. Maltseva, Lucy Stols, Michael Endres, Andrzej Joachimiak

**Affiliations:** a Center for Structural Genomics of Infectious Diseases, Consortium for Advanced Science and Engineering, University of Chicago, Chicago, IL 60667, USA; b Structural Biology Center, X-ray Science Division, Argonne National Laboratory, Argonne, IL 60439, USA; c Department of Biochemistry and Molecular Biology, University of Chicago, Chicago, IL 60367, USA

**Keywords:** SARS-CoV-2, COVID-19, macrodomain, ADP-ribose phosphatase domain, ADRP, Nsp3, Mac1, crystal structure, ADP-ribosylation

## Abstract

Among 15 nonstructural proteins (Nsps), the newly emerging Severe Acute Respiratory Syndrome coronavirus 2 (SARS-CoV-2) encodes a large, multidomain Nsp3. One of its units is the ADP-ribose phosphatase domain (ADRP; also known as the macrodomain, MacroD), which is believed to interfere with the host immune response. Such a function appears to be linked to the ability of the protein to remove ADP-ribose from ADP-ribosylated proteins and RNA, yet the precise role and molecular targets of the enzyme remain unknown. Here, five high-resolution (1.07–2.01 Å) crystal structures corresponding to the apo form of the protein and its complexes with 2-(*N*-morpholino)ethanesulfonic acid (MES), AMP and ADP-ribose have been determined. The protein is shown to undergo conformational changes to adapt to the ligand in the manner previously observed in close homologues from other viruses. A conserved water molecule is also identified that may participate in hydrolysis. This work builds foundations for future structure-based research on ADRP, including the search for potential antiviral therapeutics.

## Abstract 2

High-resolution crystal structures of the ADP-ribose phosphatase domain (ADRP; also known as the macrodomain) from SARS-CoV-2 with multiple ligands illustrate how the protein undergoes conformational changes to adapt to the ligand in the manner observed for homologues from other viruses.

## 1. Introduction   

Over the past several months, Severe Acute Respiratory Syndrome coronavirus 2 (SARS-CoV-2) has been spreading across the world, causing a disease termed COVID-19 (Coronaviridae Study Group of the International Committee on Taxonomy of Viruses, 2020 ▸). The emergence of SARS-CoV-2 and its route of viral transmission remain a mystery, but it is believed to have a zoonotic origin, likely in bats (Tang *et al.*, 2020 ▸). In late December 2019, several patients in Wuhan, People’s Republic of China were diagnosed with severe pneumonia of an unknown aetiology (Koh *et al.*, 2020 ▸; Ciotti *et al.*, 2020 ▸; Münnich *et al.*, 1988 ▸; Bogoch *et al.*, 2020 ▸). The virus has since spread rapidly around the world, infecting millions and killing hundreds of thousands (https://coronavirus.jhu.edu/map.html). These developments forced the World Health Organization to declare the outbreak a pandemic (https://www.who.int/emergencies/diseases/novel-coronavirus-2019/situation-reports). In the absence of natural community immunity, a tested vaccine or approved drugs that would help to control the epidemic, billions of people are currently under quarantine or lockdown to minimize further transmission.

The aetiological agent of COVID-19 has been isolated and was identified as a novel coronavirus resembling SARS-CoV, which was responsible for an outbreak of disease in 2002–2003 (Wu *et al.*, 2020 ▸). Like other coronaviruses, SARS-CoV-2 utilizes positive-sense RNA genome-encoded nonstructural proteins (Nsps) and structural proteins, such as spike glycoprotein (S), envelope (E), membrane (M) and nucleocapsid proteins (N), as well as accessory proteins (Wu *et al.*, 2020 ▸). Nsps are encompassed within ORF1a and ORF1ab, which produce two polyproteins, Pp1a and Pp1ab (Cui *et al.*, 2019 ▸; Kelly *et al.*, 2020 ▸). The latter protein results from a ribosomal shift that enables the continuous translation of ORF1a along with ORF1ab (Bredenbeek *et al.*, 1990 ▸). Pp1a contains two viral proteases, 3C-like main protease (Mpro, corresponding to *nsp5*) and papain-like protease (PLpro, a domain of *nsp3*), which are responsible for the post-translational processing of the two polyproteins (Snijder *et al.*, 2016 ▸). The cleavage yields 16 Nsps (15 Nsps in SARS-CoV-2) (Báez-Santos *et al.*, 2015 ▸; Thiel *et al.*, 2003 ▸) that form a large, membrane-bound replicase complex.

The largest component of the replicase assembly is Nsp3 (https://coronavirus3d.org). This multidomain protein, among other modules [the N-terminal ubiquitin-like acidic domain, SARS-unique domain, PLpro, nucleic acid binding (NAB) domain, transmembrane domain and Y-domain; reviewed in Lei *et al.* (2018) ▸], contains the ADP-ribose phosphatase domain (ADRP; also known as the macrodomain). ADRP was identified 30 years ago by bio­informatics as a unique and conserved domain, initially termed the X-domain (Lee *et al.*, 1991 ▸), that was found in the genomes of the *Togaviridae*, *Coronaviridae* and *Hepeviridae* families. Since then it has been also discovered in the *Iridiviridae*, *Poxviridae* and *Myoviridae*, which includes phages. The first crystallographic model of an ADRP (Saikatendu *et al.*, 2005 ▸) enabled the classification of the protein as a member of the macroH2A-like family. The founding member of this family is a large, nonhistone part of the histone macroH2A, known as the macrodomain (Pehrson & Fried, 1992 ▸). To date, the structurally characterized viral ADRPs comprise 11 representatives, including those from SARS-CoV, MERS-CoV (Middle East Respiratory Syndrome virus), H-CoV-229E (Human coronavirus 229E) and others.

The nonviral macrodomains have been shown to recognize ADP-ribose (ADPr) in the free form and in macromolecule-linked forms, as well as attached to other ligands. In addition to binding, some macrodomains possess catalytic activities, including the removal of ADPr from ADP-ribosylated proteins or nucleic acids (DNA and RNA) (Munnur *et al.*, 2019 ▸). ADP-ribosylation is a regulatory modification that is present in all kingdoms of life and is known to play a role in DNA damage repair, signal transduction, the immune response and other cellular stresses (for a review, see, for example, Crawford *et al.*, 2018 ▸). The appendage is transferred onto the target by ADP-ribosyl transferases (ARTs), which are classified as either diphtheria toxin-like enzymes [ARTDs; previously known as poly(ADP-ribose)polymerases or PARPs] or cholera toxin-like enzymes (ARTCs). Some sirtuins also carry out such reactions. Both groups of proteins utilize NAD^+^ as an ADPr donor. ARTDs catalyze the transfer of either single (mono-ADP-ribosylation, MARylation) or multiple (poly-ADP-ribosylation, PARylation; mostly PARP1 and PARP2) ADPr units, primarily onto glutamate/aspartate residues, but sometimes also onto serine residues. In nucleic acids, the modification is attached to a phosphoryl group at the terminal end of DNA/RNA. ARTCs only carry out MAR­ylation and preferentially act on arginine residues. De-ADP-ribosylation requires several enzymes. The polymeric fragment of the modification is removed by poly(ADP-ribosyl)glycohydrolase (PARG), while the final ADP-ribose unit is cleaved from glutamate/aspartate residues by the macrodomain. Enzymes from the (ADP-ribosyl) hydrolase (ARH) family have specificity for serine and arginine cargos (Fontana *et al.*, 2017 ▸; Moss *et al.*, 1988 ▸). The released ADPr is used in recycling pathways.

Currently, six classes of macrodomains have been distinguished: MacroH2-like, AlC1-like, PARG-like, Macro2-type, SUD-M-like (also known as Mac2/Mac3) and MacroD-type (Rack *et al.*, 2016 ▸). These categories are derived from structural similarities rather than from sequence similarity. Most viral macrodomains fall into the MacroD-like family, which encompasses the human homologues MacroD1 and MacroD2, and is associated with the removal of mono(ADP-ribosyl­ation). *In vivo* experiments have shown that viral MacroD-like macrodomains can hydrolyze ADPr-1′′-phosphate, but the catalytic efficiency of this process has raised doubts about its physiological implications (Egloff *et al.*, 2006 ▸). Instead, it has been suggested that these macrodomains might play roles analogous to MacroD1 and MacroD2. Indeed, de-ADP-ribosylating activities on proteins and RNA, including the removal of the entire PAR chain, have been demonstrated for several viral macrodomains, for example those from SARS-CoV and H-CoV-229E (Li *et al.*, 2016 ▸; Eckei *et al.*, 2017 ▸; Munnur *et al.*, 2019 ▸). Binding to PAR (Egloff *et al.*, 2006 ▸) and RNA (Malet *et al.*, 2009 ▸) has also been reported. Importantly, the wide range of affinities and activities practically prevents similarity-based assumptions about the physiological roles of these proteins.

On the physiological level, such biochemical activity means that the role of ADRP would be to counteract the function of ARTD/PARP proteins. The latter enzymes are upregulated by interferon, indicating their relevance in the innate immune response. Systematic knockdown studies of all 17 mammalian PARPs in a Mouse Hepatitis Virus model with macrodomain mutants implicated PARP12 and PARP14 in the control of virus replication (Grunewald *et al.*, 2019 ▸). Interestingly, PARP12, which is able to auto-ADP-ribosylate, belongs to a family of zinc-finger CCCH domains that are known to bind to RNAs, including those of viral origin. The antiviral properties of the protein have been linked to its enzymatic activity, its colocalization with polyribosomes via an RNA-binding domain and its interference with translation machinery (Atasheva *et al.*, 2014 ▸). Also, PARP10, which is known to modify RNA (Munnur *et al.*, 2019 ▸), has been shown to inhibit viral replication (Atasheva *et al.*, 2012 ▸, 2014 ▸). The role of ADRP in jeopardizing the immune response has also been emphasized by studies showing that viruses with mutated macrodomains replicated poorly in bone-marrow-derived macrophages, which are the primary cells involved in mounting the innate immune response (Grunewald *et al.*, 2019 ▸). Along the same lines, viruses with deactivated macrodomains were sensitive to interferon pretreatment (Kuri *et al.*, 2011 ▸). It has recently been proposed that de-mono-ADP-ribosylation of STAT1 by ADRP may be linked to the Cytokine Storm Syndrome that is commonly observed in severe cases of COVID-19 (Claverie, 2020 ▸).

Since the role of macrodomains in pathogenesis is essential, it appears that their inhibition may help to reduce the viral load and facilitate recovery. Therefore, these proteins might be attractive targets for the development of small-molecule antivirals, assuming that highly selective compounds could be found that discriminate between viral and human macrodomains (Virdi *et al.*, 2020 ▸). As a step towards this goal, we have determined the crystal structure of SARS-CoV-2 ADRP in multiple states: in the apo form and in complexes with 2-(*N*-morpholino)ethanesulfonic acid (MES), AMP and ADPr. With the apo crystals diffracting to atomic resolution, we have developed a robust system for structure-based experiments to identify potential small-molecule inhibitors.

## 2. Materials and methods   

### 2.1. Gene cloning, protein expression and purification   

The gene for ADRP was synthesized using a codon-optimization algorithm for *Escherichia coli* expression and was cloned into a pET-15b vector (Bio Basic) and transformed into the *E. coli* BL21(DE3) Gold strain (Stratagene). For preparative purposes, for each protein batch a 4 l culture of LB Lennox medium was grown at 37°C (190 rev min^−1^) in the presence of 150 µg ml^−1^ ampicillin. Once the culture reached an OD_600_ of ∼1.0, the temperature setting was changed to 4°C. When the bacterial suspension had cooled to 18°C it was supplemented with the following components at the indicated concentrations: 0.2 m*M* isopropyl β-d-1-thiogalactopyranoside, 0.1% glucose and 40 m*M* K_2_HPO_4_. The temperature was set to 18°C for a 20 h incubation. The bacterial cells were harvested by centrifugation at 7000*g* and the cell pellets were collected.

We have developed two protocols for purification, differing in the buffer composition. For the first batch of protein [ADRP(b1)] HEPES–NaOH pH 8.0 was used as the primary buffering component, while subsequent purifications [ADRP(b2)] used Tris–HCl at an identical pH value, unless stated otherwise. All of the steps were the same and are described below. The cell pellets were resuspended in 12.5 ml lysis buffer [500 m*M* NaCl, 5%(*v*/*v*) glycerol, 50 m*M* HEPES (or Tris) pH 8.0, 20 m*M* imidazole with 10 m*M* β-mercapto­ethanol in Tris-based purification] per litre of culture and sonicated at 120 W for 5 min (4 s on, 20 s off). The insoluble material was removed by centrifugation at 30 000*g* for 1 h at 4°C. The supernatant was mixed with 3 ml Ni^2+^ Sepharose (GE Healthcare Life Sciences) equilibrated in lysis buffer with the imidazole concentration increased to 50 m*M*, and the suspension was applied onto a Flex-Column (Kimble; catalogue No. 420400-2510) connected to a Vac-Man vacuum manifold (Promega). Unbound protein was washed out by controlled suction with 160 ml lysis buffer (50 m*M* imidazole). The bound protein was eluted with 15 ml lysis buffer supplemented to 500 m*M* imidazole pH 8.0. 2 m*M* dithiothreitol was added and the protein was subsequently treated overnight at 4°C with Tobacco Etch Mosaic Virus (TEV) protease at a 1:20 protease:protein ratio. The protein solution was concentrated using a 10 kDa molecular-weight cutoff filter (Amicon-Millipore) and was further purified on a Superdex 200 size-exclusion column in lysis buffer in which the β-mercapto­ethanol had been replaced by 1 m*M* tris(2-carboxy­ethyl)phosphine (TCEP). The fractions containing ADRP were pooled and run one more time through Ni^2+^ Sepharose. The flowthrough was collected and buffer-exchanged into crystallization buffer [150 m*M* NaCl, 20 m*M* HEPES pH 7.5 (or Tris pH 8.0), 1 m*M* TCEP] via tenfold concentration and dilution repeated three times. The protein was immediately used in crystallization trials. The final concentration of ADRP(b1) was 22 mg ml^−1^ and the final concentration of ADRP(b2) was 32 mg ml^−1^.

### 2.2. Crystallization   

Crystallization screening was performed by the sitting-drop vapour-diffusion method in 96-well CrystalQuick plates (Greiner Bio-One). The plates were set up with a Mosquito liquid dispenser (TTP Labtech) utilizing 400 nl of purified protein sample, which was mixed with 400 nl of well solution and equilibrated against 135 nl of reservoir solution. ADRP(b1) was used to grow apo-form crystals and for crystallization with AMP and ADPr. The AMP complex was prepared by adding AMP (pH 6.5) to a final concentration of 12 m*M*. To obtain the ADPr complex, the protein was mixed with ADPr in a 1:2 molar ratio. Crystallization screening was performed using the MCSG1, MCSG4 (Anatrace), SaltRX (Hampton Research), PACT Suite (Qiagen) and Index (Hampton Research) screens. ADRP(b2) was set up at 18 mg ml^−1^ with the Pi-minimal (Jena Biosciences), Protein Complex Suite (Qiagen) and Index (Hampton Research) screens. In all cases, the plates were incubated at 289 K.

ADRP(b1) crystals grew from a condition consisting of 0.1 *M* CHES pH 9.5, 30%(*w*/*v*) PEG 3000, yielding the structure denoted ADRP-APO1. The complex with ADPr was obtained from 0.01 *M* sodium citrate, 33% PEG 6000, giving the structure labelled ADRP–ADPr. The complex with AMP was grown from 0.1 *M* MES pH 6.5, 30%(*w*/*v*) PEG 4000, giving the structure labelled ADRP–AMP. ADRP(b2) crystals grew from 0.1 *M* MES pH 6.5, 30%(*w*/*v*) PEG 4000, yielding the ADRP–MES complex, and from 30 m*M* sodium/potassium tartrate, 150 m*M* AMPD–Tris pH 9.0, 34.3%(*w*/*v*) PEG 5000 MME, giving the crystals labelled ADRP-APO2.

### 2.3. Data collection, structure determination and refinement   

Prior to flash-cooling in liquid nitrogen, the crystals were cryoprotected in their mother liquor supplemented with either an increased concentration of PEG 3000 up to 40% (ADRP-APO1), 5% glycerol (ADRP–ADPr), 7% ethylene glycol (ADRP–AMP) or 10% ethylene glycol (ADRP–MES). The ADRP-APO2 crystals did not require cryoprotection. The X-ray diffraction experiments were carried out at 100 K on the Structural Biology Center 19-ID beamline at the Advanced Photon Source, Argonne National Laboratory. The diffraction images were recorded on a PILATUS3 X 6M detector. The data set was processed and scaled with the *HKL*-3000 suite (Minor *et al.*, 2006 ▸). Intensities were converted to structure-factor amplitudes using *TRUNCATE* (French & Wilson, 1978 ▸; Padilla & Yeates, 2003 ▸) from the *CCP4* package (Winn *et al.*, 2011 ▸). The ADRP-APO1 structure was determined by molecular replacement (MR) using *MOLREP* (Vagin & Teplyakov, 2010 ▸) as implemented in the *HKL*-3000 software package with the SARS-CoV ADRP structure (PDB entry 2acf; Saikatendu *et al.*, 2005 ▸) as a search probe. The subsequent structures were solved by MR using the refined SARS-CoV-2 ADRP structure as a model. In all cases, the initial solution was manually adjusted using *Coot* (Emsley *et al.*, 2010 ▸) and then iteratively refined using *Coot*, *Phenix* (Liebschner *et al.*, 2019 ▸) and *REFMAC* (Murshudov *et al.*, 2011 ▸; Winn *et al.*, 2011 ▸). The final rounds of refinement were carried out in *Phenix* (ADRP-APO1, ADRP–ADPr and ADRP–MES) or *REFMAC* (ADRP–AMP and ADRP-APO2). The ADRP-APO1 and ADRP–ADPr structures were refined with TLS parameterization of anisotropic displacement parameters, while for the remaining structures a full anisotropic refinement was calculated. The same 5% of reflections were excluded throughout refinement (in both the *REFMAC* and *Phenix* refinements). The final models show nearly complete polypeptide chains. The residues that were not modelled owing to a lack of interpretable electron density include Gly1-Glu2 and Glu170 in chains *A* and *B* for ADRP-APO1; Gly1-Glu2-Val3 and Leu169-Glu170 in chain *A*, and Gly1-Glu2 and Glu170 in chain *B* for ADRP–ADPr; Gly1-Glu2 in chain *A*, and Gly1-Glu2 and Glu170 in chain *B* for ADRP–AMP; Gly1-Glu2-Val3 and Glu170 for ADRP–MES; and Gly1-Glu2 for ADRP-APO2. The stereochemistry of the structure was checked with *MolProbity* (Chen *et al.*, 2010 ▸), *PROCHECK* (Laskowski *et al.*, 1993 ▸) and the Ramachandran plot, and was validated with the PDB Validation Server. The data-collection and processing statistics are given in Table 1 ▸. The atomic coordinates and structure factors have been deposited in the PDB under accession codes 6vxs, 6w02, 6w6y, 6wcf and 6wen.

## 3. Results and discussion   

### 3.1. Protein production and structure determination   

We used an *E. coli* codon-optimized synthetic gene with a sequence corresponding to SARS-CoV-2 ADRP to produce the protein for crystallographic and biochemical studies. The protein was crystallized under several conditions, yielding five crystal structures, denoted ADRP-APO1 (apo form), ADRP–ADPr (complex with ADPr), ADRP–AMP (complex with AMP), ADRP–MES [complex with 2-(*N*-morpholino)ethanesulfonic acid] and ADRP-APO2 (apo form). The ADRP-APO1 structure was solved first by molecular replacement using the SARS-CoV homologue structure (PDB entry 2acf; Saikatendu *et al.*, 2005 ▸) as a search model. All of the subsequent structures were solved by MR using the refined SARS-CoV-2 ADRP structure as a template.

ADRP-APO1 was refined to 2.01 Å resolution. The protein crystallized in space group *P*1, with two molecules in the unit cell. None of the polypeptides contains ligand in the catalytic pocket, but there is an *N*-cyclohexyl-2-aminoethanesulfonic acid (CHES) molecule bound on the surface. Like ADRP-APO1, the ADRP–ADPr structure was solved in space group *P*1. It was refined with reflections extending to 1.50 Å resolution, although 88% completeness was only achieved to 1.65 Å resolution. The ADPr ligand is well defined in the electron-density map in both polypeptide chains. ADRP–AMP crystallized in space group *P*2_1_, also with two molecules in the asymmetric unit. The atomic model was refined to 1.45 Å resolution. In the ADPr-binding pocket, one of the protein molecules (chain *A*) binds an AMP ligand with occupancy 0.8, while the other (chain *B*) binds a MES molecule with occupancy 0.7. In the latter case, there is additional electron density in the position where the adenine ring binds, but its quality prevented an acceptable interpretation. The ADRP–MES crystals also belonged to space group *P*2_1_, but with a smaller unit cell and with only one protein molecule in the asymmetric unit. These crystals diffracted to 1.07 Å resolution. Two MES molecules were identified in the structure: one in the ADPr-binding pocket and another on the protein surface. Finally, the ADRP-APO2 structure was determined in space group *C*2, with one protein chain in the asymmetric unit. We used reflections extending to 1.35 Å resolution in refinement. The binding pocket in ADRP-APO2 has no small molecule present, with the exception of solvent. In all structures the polypeptide chains are nearly complete, with only a few residues missing at the termini, as detailed in Section 2. The data-collection and structure-refinement statistics are given in Table 1 ▸. All of the structures have been deposited in the Protein Data Bank (PDB).

### 3.2. Overall structure   

**Figure 1:**
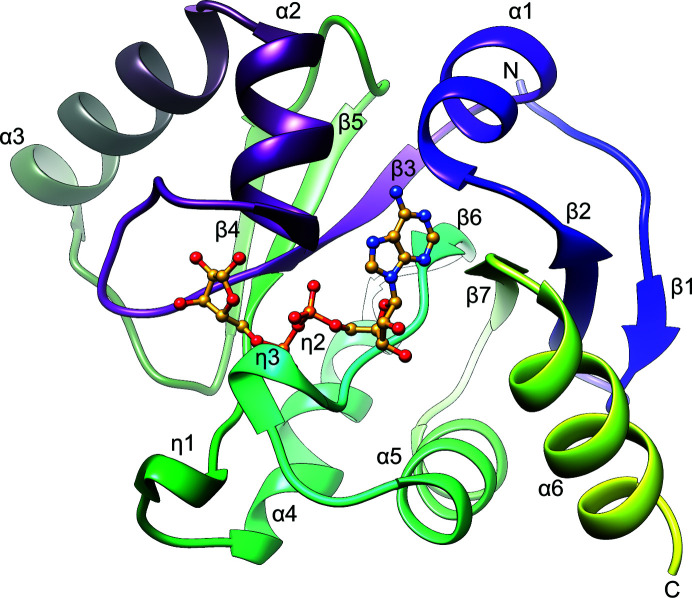
The structure of SARS-CoV-2 ADRP. The ribbon diagram shows ADRP-APO2. The ADPr ligand molecule is shown based on superposition with the ADRP–ADPr structure.

The structure of SARS-CoV-2 ADRP features a central seven-stranded mixed β-sheet (β1↑, β2↓, β7↓, β6↓, β3↓, β5↓, β4↑) sandwiched between two layers of helices: α1, α2 and α3 on one side and η1, α4/η2, η3, α5 and α6 on the other (Fig. 1 ▸). These features follow the previously established characteristic fold of a MacroD-like macrodomain as described previously for several viral homologues. According to *DALI* calculations (Holm & Rosenström, 2010 ▸), the closest structural relative is from SARS-CoV (PDB entry 2acf; *Z*-score of 33.9 and r.m.s.d. of 0.5 Å over 168 C^α^ atoms superposed onto ADRP-APO2; Saikatendu *et al.*, 2005 ▸). This homologue shares 71% sequence identity and 82% similarity with the SARS-CoV-2 ADRP (as determined by *EMBOSS Needle*; Rice *et al.*, 2000 ▸). The next hit corresponds to the MERS-CoV homologue (PDB entry 5hih, *Z*-score of 28.0 and r.m.s.d. of 1.3 Å over 163 C^α^ atoms; Lei & Hilgenfeld, 2016 ▸), which displays 40% sequence identity and 61% similarity. Subsequent neighbours with r.m.s.d.s of up to 2.0 Å include the homologues from *Tylonycteris* bat coronavirus HKU4 (PDB entry 6men; R. G. Hammond, N. Schormann, R. L. McPherson, A. K. L. Leung, C. C. S. Deivanayagam & M. A. Johnson, unpublished work), feline coronavirus (FIP; PDB entry 3ew5; Wojdyla *et al.*, 2009 ▸), H-CoV-229E (PDB entry 3ejg; Piotrowski *et al.*, 2009 ▸) and H-CoV-NL63 (PDB entry 2vri; Y. Piotrowski, J. R. Mesters, R. Moll & R. Hilgenfeld, unpublished work).

The SARS-CoV-2 ADRP structures show a high level of agreement amongst each other. The r.m.s.d.s for ADRP-APO2 superposition range from 0.3 and 0.4 Å for ADRP–AMP through 0.4 and 0.5 Å for ADRP–ADPr and ADRP–MES up to 0.6 and 0.7 Å for ADRP-APO1.

### 3.3. Substrate-binding pocket   

**Figure 2:**
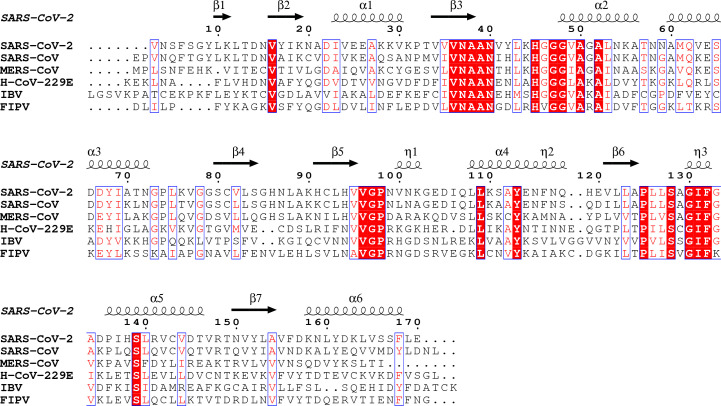
Sequence alignment of SARS-CoV-2 ADRP homologues from coronaviruses with structures of complexes with ADPr available in the PDB: SARS-­CoV-­2 (PDB entry 6wen, chain *A*), SARS-CoV (PDB entry 2fav, chain *A*), MERS-CoV (PDB entry 5dus, chain *A*), H-CoV-229E (PDB entry 3ewr, chain *A*), IBV (PDB entry 3ewp, chain *A*) and FIPV (PDB entry 3jzt, chain *A*). The secondary-structure elements are labelled for SARS-CoV-2 ADRP.

The well defined substrate-binding pocket is created by the C-terminal edges of the central β-strands β3, β5, β6 and β7 and the surrounding fragments, primarily loop β3–α2, the N-terminus of helix α1 and a long loop connecting β6 to α5, which contains the short 3_10_-helix η3. These elements encompass four conserved sequence motifs (Fig. 2 ▸) that are shared by the family members (Saikatendu *et al.*, 2005 ▸). The first such block is present at the end of β3 and is followed by another that extends into helix α2. The third segment corresponds to the end of β5 and the last segment overlaps with helix η3.

**Figure 3:**
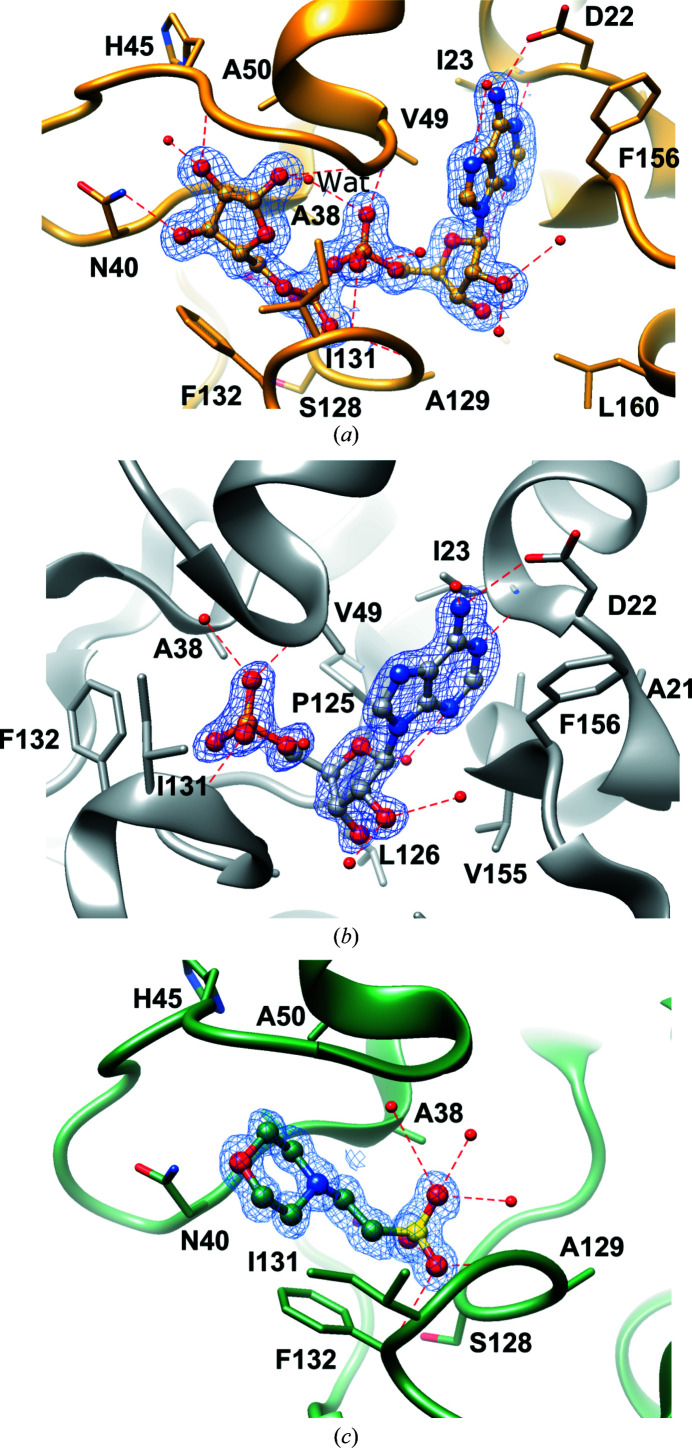
Ligand binding in SARS-CoV-2 ADRP. (*a*) ADPr binding (PDB entry 6w02, chain *A*). (*b*) AMP binding (PDB entry 6w6y, chain *A*). (*c*) MES binding (PDB entry 6wcf). All 2*mF*_o_ − *DF*_c_ electron-density maps are contoured at the 1.2σ level.

Within the crevice, four sections can be distinguished, corresponding to adenine-binding, distal ribose-binding, diphosphate-binding and proximal ribose-binding sites, denoted here as A, R1, P1-P2 and R2, respectively. The ADRP/ADPr structure illustrates how the ligand molecule interacts with these subsites (Figs. 3 ▸ and 4 ▸). The adenine moiety is sandwiched between α2 and β7 in a mostly hydrophobic environment created by Ile23, Val49, Pro125, Val155 and Phe156. Polar contacts are facilitated by Asp22, which forms a hydrogen bond to the N6 atom via its carboxylate group, and by the main-chain amide of Ile23, which binds to the N1 atom. In addition, water-mediated contacts link the N3 atom to the main chain of Ala154 and Leu126. The A site has limited sequence conservation: only Pro125 and Asp22 are conserved among the homologues. Other hydrophobic residues are replaced by side chains with a similar chemical character. The striking exception is Phe156, which is replaced by Asn in the closest homologues from SARS-CoV and MERS-CoV. In other viral representatives it is substituted by another hydrophobic residue. The distal ribose ring only participates in water-mediated hydrogen bonds to the main-chain amide of Leu126 and the carbonyl group of Ala154 via the ring O atom and to the Asp157 main chain and side chain via the OH2′ group. The diphosphate moiety binds between two loops, β3–α2 and β6–(η3)–α5, that cover three segments with high sequence conservation, including a glycine-rich segment (Gly46-Gly47-Gly48) within the former loop. Here, the ligand forms direct hydrogen bonds to the main-chain amides of Val49, Ser128, Gly130, Ile131 and Phe132 and water-mediated contacts with Ala38, Ala39, Ala50, Val95 and Gly97. An elaborate network of water molecules also links the diphos­phate to Gly47, Ala129 and Asp157. Finally, the proximal ribose ring is stabilized in the pocket by hydrophobic interactions with Phe132 and Ile131, as well as a set of hydrogen bonds with Gly46 (OH2′), Gly48 (OH1′) and Asn40 (OH3′). All of these residues are conserved. Additional bonds to the main-chain peptides of Asn40, Lys44 and Ala50 are water-mediated. Interestingly, as described above, only a few hydrogen bonds involve protein side chains, with most such contacts utilizing main-chain atoms. This may explain why there is less pressure on amino-acid sequence preservation, since main-chain interactions can be accomplished with multiple side-chain combinations.

**Figure 5:**
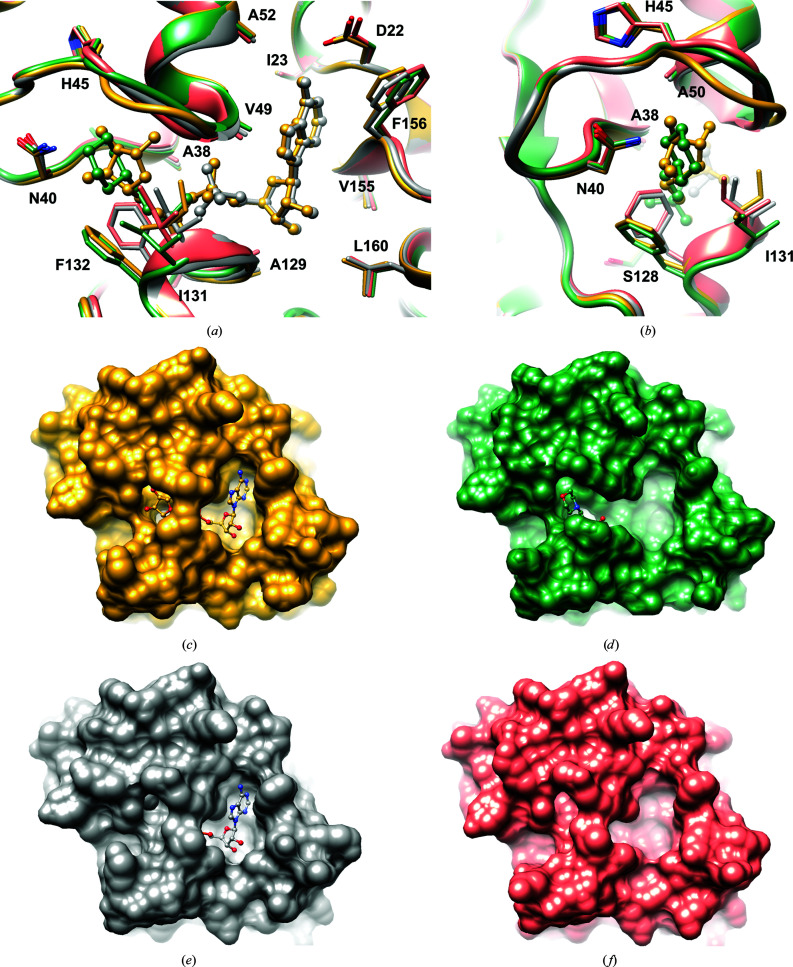
Ligand-induced conformational changes in the SARS-CoV-2 ADRP structure. (*a*) Superposition of the ADRP–ADPr complex (yellow; PDB entry 6w02, chain *A*) with ADRP–AMP (grey; PDB entry 6w6y, chain *A*), ADRP–MES (green; PDB entry 6wcf) and ADRP-APO2 (coral; PDB entry 6wen). The ligand molecules are shown in ball-and-stick representation. (*b*) As in (*a*), but rotated ∼90°. (*c*) Surface representation of the ADRP–ADPr complex. (*d*) Surface representation of the ADRP–MES complex. (*e*) Surface representation of the ADRP–AMP complex. (*f*) Surface representation of the ADRP-APO2 structure.

Similar contacts are observed in the ADRP–AMP structure (Fig. 3 ▸), in which the ligand superposes well with the AMP portion of the ADPr ligand (Fig. 5 ▸). The ADRP–MES complex, however, presents a somewhat different scenario, in which the 2-*N*-morpholine ring takes the place of the proximal ribose and a sulfonic acid substitutes for the distal phosphate. The latter group forms the hydrogen bonds observed in the ADPr complex and an additional network of solvent-facilitated contacts. The ring moiety appears to primarily be anchored by hydrophobic interactions with Phe132 and Ile131, and a hydrogen bond might potentially be present between the morpholine O atom and Asn40, although the geometry is rather unfavourable.

### 3.4. Ligand-induced conformational changes   

While the interactions with ligands do not trigger major conformational changes in the overall structure, significant shifts are observed in the binding pocket itself. This is consistent with the differential scanning fluorimetry (DSF) measurements, which show that AMP and ADP do not affect the thermal stability of ADRP and only ADPr causes a small (2.5°C) increase in *T*_m_ (Supplementary Fig. S1). Superpositions of the apo forms with the complexed proteins indicate several adjustments (Fig. 5 ▸). Firstly, in the A site Phe156 is brought closer to the pocket lumen when it is occupied by the nucleotide, as seen in the ADRP–ADPr and ADRP–AMP complexes. The glycine-rich β3–α2 loop shows a high degree of flexibility, with roughly the same geometry but slightly different positions in ADRP-APO1, ADRP–MES, ADRP–AMP and ADRP-APO2 (Fig. 5 ▸). In the latter structure, however, the Gly46-Gly47 peptide bond also has an alternative conformation. A significant change is observed in ADRP–ADPr, where the loop has to rearrange to make the main-chain amide N atoms accessible for interactions with the ribose OH1′ and OH2′ groups. Finally, the geometry of the β6–(η3)–α5 loop and the rotameric states of Phe132 and Ile131, contributing to the P1-P2 and R2 sites, also adapt depending on the ligand identity. The apo and AMP-bound forms contain the η3 element within the β6–α5 linker, while in the ADPr and MES complexes this region does not observe 3_10_-helix parameters. The primary reason for this is the flipping of the Ala129-Gly130 peptide bond, which in the absence of phosphate 2, or its mimetic, has the carbonyl group facing the P2 site. Otherwise, with P2 occupied, the Gly130 amide group is hydrogen-bonded to the ligand, as described above. Ile131 and Phe132 are also observed in two states. With the R2 pocket empty or containing MES, Ile131 adopts the pt rotamer (p, plus, centred near +60°; t, trans, centred near 180°), while in the presence of a ribose ring it converts to the mt state (m, minus, centred near −60°) (Hintze *et al.*, 2016 ▸). Phe132 follows a somewhat similar pattern: in the first scenario it adopts an m-10 conformation, while in the latter it adopts an m-80 conformation. These rearrangements are necessary to provide sufficient room for the ligand and proper interactions. Similar transformations in the ligand-binding pocket have been reported for other homologues (Egloff *et al.*, 2006 ▸; Piotrowski *et al.*, 2009 ▸; Wojdyla *et al.*, 2009 ▸). In the ADRP-APO1 structure, while the described geometry of the β6–η3–α5 linker remains similar to that in ADRP-APO2, the entire section and the neighbouring η1 are shifted away from the binding pocket.

### 3.5. Similarity of ADPr binding between ADRP homologues   

**Figure 6:**
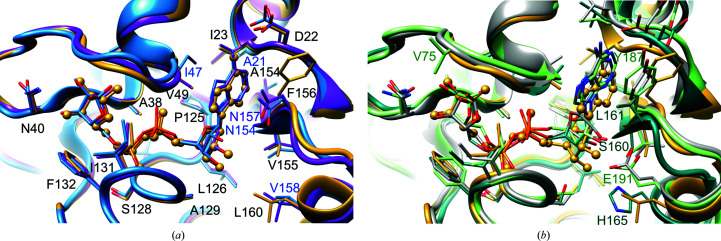
Comparison of SARS-CoV-2 ADRP–ADPr with homologous complexes. (*a*) Superposition of SARS-CoV-2 ADRP–ADPr (yellow) with homologues from MERS-CoV (PDB entry 5dus; blue) and SARS-CoV (PDB entry 2fav; purple). (*b*) Superposition of SARS-CoV-2 ADRP–ADPr with homologues from H-CoV-229E (PDB entry 3ewr; grey), IBV (PDB entry 3ewp; teal) and FIPV (PDB entry 3jzt; green). In (*a*), SARS-CoV-2 residues are labelled in black. In both panels, selected residues of homologous proteins are labelled.

The PDB currently contains four other coronaviral ADRPs in complexes with ADPr, from SARS-CoV (PDB entry 2fav; Egloff *et al.*, 2006 ▸), MERS-CoV (PDB entries 5hol and 5dus; Lei *et al.*, 2018 ▸; Cho *et al.*, 2016 ▸) and H-CoV-229E (PDB entry 3ewr; Xu *et al.*, 2009 ▸), and also those from the animal-infecting Infectious Bronchitis Virus (IBV; PDB entry 3ewp; Piotrowski *et al.*, 2009 ▸) and Feline Infectious Peritonitis Virus (FIPV; PDB entry 3jzt; Wojdyla *et al.*, 2009 ▸). The SARS-CoV and MERS-CoV complexes mostly follow the pattern of inter­actions observed in the current structure (Fig. 6 ▸). The ligand geometry is also preserved. The elements that are distinct are located in the A and R1 sites. Most strikingly, Phe156 in the SARS-CoV-2 ADRP is replaced by Asn157 in the SARS-CoV homologue (Asn154 in MERS-CoV in PDB entry 5dus) that stacks against the adenine ring and at the same time creates water-mediated hydrogen bonds to the distal ribose. Three other sequence discrepancies with the MERS-CoV ADRP are located in this region: Ile23 is replaced by Ala21 (Ile24 in SARS-CoV), Val49 by Ile47 (Val50 in SARS-CoV) and Leu160 by Val158 (Leu161 in SARS-CoV). These changes are most likely to be responsible for a small discrepancy between the ADPr molecules bound to these structures.

A more divergent picture is observed in the distant homologues from H-CoV-229E, IBV and FIPV (Fig. 6 ▸), mainly in the A and R1 sites, with the caveat that the distal ribose in the H-CoV-229E ADRP complex has the wrong stereochemistry. In these homologues, we observe sequence variation in the Phe156 position, which is replaced by other hydrophobic residues. The adenine ring is significantly shifted with respect to SARS-CoV-2 ADRP. The interaction between the N1 atom of adenine and the Asp22 equivalent is lost, even though the latter amino acid is conserved in the three-dimensional context (Asp20 in IBV does not overlap in the primary sequence). The distal ribose is better anchored in place: hydrogen bonds link it either to the glutamate residue (Glu156 in H-CoV-229E and Glu191 in FIPV) that substitutes Leu160 or to the serine in the position of Val155 (Ser160 in IBV). Another notable difference is observed in the R2 site, where the equivalents of Ile131 in the H-CoV-229E and IBV proteins adopt outlier rotamers, yet the electron-density maps allow the more favourable conformations seen in our structure to be modelled. In these two models, the proximal ribose adopts an α configuration of the anomeric C atom (Fig. 6 ▸). Such a state, with partial occupancy, has also been reported for one of the SARS-CoV complexes (Egloff *et al.*, 2006 ▸) and is linked to the alternative, apo-like conformation of Gly47-Gly48. The α configuration is most likely to illustrate the geometry of the putative substrate, as only then is the hydroxyl group exposed to the solvent, providing room for the macromolecule portion of the substrate.

The common feature in the R2 site among all homologues is the presence of equivalents of Phe132, Asn40 and the glycine-rich loop: these elements have been shown to be crucial for ADRP activity of the SARS-CoV protein through mutational studies (Egloff *et al.*, 2006 ▸; Li *et al.*, 2016 ▸) and the study of macrodomains from viruses from other families (Malet *et al.*, 2009 ▸; Li *et al.*, 2016 ▸).

### 3.6. Catalytic mechanism   

In the absence of potential catalytic residues that are conserved across all of the macrodomains, Jankevicius and coworkers proposed an enzymatic mechanism involving substrate-assisted catalysis, in which a water molecule that is responsible for nucleophilic attack on the anomeric C atom of the ribose is activated by the Pα group (Jankevicius *et al.*, 2013 ▸). In the current ADRP–ADPr structure, the candidate water molecule (Wat) binds to the amide group of Ala50, the carbonyl of Ala38, the O atom of Pα and the OH1′ group of the proximal ribose ring of ADPr (Figs. 3 ▸ and 4 ▸). In the ADRP-APO2 and ADRP–MES structures, the last hydrogen bond is replaced by an interaction with the carbonyl group of Gly47, enhancing the proton-abstraction capabilities of the environment. Presumably, based on the models in which ADPr exists as an α anomer, a similar network would be likely to occur in the complex with ADPr protein or RNA substrates, assuming no major conformational rearrangements. The water molecule is ideally located to pursue a nucleophilic attack on the anomeric C atom.

## 4. Conclusions   

The large, multidomain Nsp3 includes an ADP-ribose phosphatase domain (ADRP/MacroD), which is believed to interfere with the host immune response by removing ADP-ribose from ADP-ribosylated proteins or RNA. Our study presents five atomic and high-resolution structures of SARS-CoV-2 ADRP, including the apo form and complexes with MES, AMP and ADPr. Their analysis shows that the enzyme undergoes conformational changes upon ADPr binding, which is in agreement with several previous reports showing such rearrangements. The shifts, which affect both the main chain and side chains, are observed primarily around the proximal ribose, where the protein has to make room for the sugar moiety and adjust to both configurations of the anomeric C atom. The active-site water molecule is proposed to carry out a nucleophilic attack on the anomeric C atom of the ribose. Our high-resolution studies of ADRP complexes with ligands allow accurate modelling of the active site of ADRP and will aid in the design of compounds that can inhibit the activity of this enzyme.

## 5. Related literature   

The following reference is cited in the supporting information for this article: Huynh & Partch (2015 ▸).

## Supplementary Material

PDB reference: SARS-CoV-2 ADP-ribose phosphatase, apo, 6vxs

PDB reference: 6wen

PDB reference: complex with ADP-ribose, 6w02

PDB reference: complex with AMP, 6w6y

PDB reference: complex with MES, 6wcf

Supplementary Figure S1. DOI: 10.1107/S2052252520009653/lz5040sup1.pdf

## Figures and Tables

**Figure 4:**
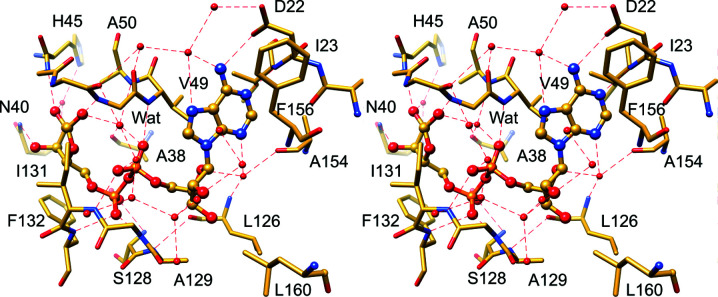
Stereoview of ADPr binding in the SARS-CoV-2 ADRP binding site.

**Table 1:** Data-processing and refinement statistics The values in parentheses are for the highest resolution shell. For ADRP–ADPr, the values for another shell in which the completeness achieves ∼90% are also given.

Structure	ADRP-APO1	ADRP–ADPr	ADRP–AMP	ADRP–MES	ADRP-APO2
Data processing					
Wavelength (Å)	0.9792	0.9792	0.9792	0.9792	0.9792
Resolution range (Å)	50.00–2.01 (2.04–2.01)	50.00–1.50 (1.69–1.65/1.53–1.50)	50.00–1.45 (1.48–1.45)	50.0–1.07 (1.09–1.07)	50.00–1.35 (1.37–1.35)
Space group	*P*1	*P*1	*P*2_1_	*P*2_1_	*C*2
*a*, *b*, *c* (Å)	30.39, 37.90, 65.40	30.27, 37.84, 68.30	37.75, 33.38, 121.15	37.17, 33.18, 60.62	139.67, 29.68, 37.89
α, β, γ (°)	84.37, 82.11, 90.11	97.86, 97.38, 89.94	90.00, 95.09, 90.00	90.00, 96.11, 90.00	90.00, 103.52, 90.00
Unique reflections, merged	16545 (820)	42236 (2285/941)	52864 (2626)	64283 (3077)	32168 (1515)
Multiplicity	5.3 (3.4)	3.4 (3.5/2.9)	5.3 (4.6)	5.9 (3.9)	6.2 (2.7)
Completeness (%)	90.8 (91.2)	80.8 (87.7/36.1)	98.6 (99.5)	97.9 (94.8)	95.7 (91.4)
Mean *I*/σ(*I*)	8.44 (3.76)	25.3 (11.1/5.98)	21.3 (1.50)	18.7 (1.46)	24.13 (2.65)
Wilson *B* factor (Å^2^)	23.07	15.93	25.77	10.00	
*R*_merge_ †	0.221 (0.373)	0.075 (0.145/0.224)	0.109 (1.249)	0.094 (1.110)	0.089 (0.483)
CC_1/2_ ‡	0.971 (0.804)	0.982 (0.973/0.935)	0.990 (0.540)	0.995 (0.564)	0.994 (0.747)
Refinement					
Resolution range (Å)	37.71–2.03	37.48–1.50	40.26–1.45	33.11–1.07	36.86–1.35
Reflections (work/test)	15779/770	39680/2116	47600/2627	60978/3250	30573/1595
*R*_work_/*R*_free_ §	0.186/0.234	0.150/0.173	0.142/0.189	0.125/0.15	0.104/0.144
No. of non-H atoms					
Total	2854	3016	2906	1634	1686
Macromolecules	2526	2567	2571	1413	1385
Ligands/solvent	50/278	76/373	35/300	24/197	1/300
Protein residues	334 [2 chains]	332 [2 chains]	335 [2 chains]	165 [1 chain]	168 [1 chain]
R.m.s.d., bond lengths (Å)	0.003	0.005	0.012	0.012	0.012
R.m.s.d., angles (°)	0.513	0.803	1.683	1.407	1.659
Ramachandran favoured¶ (%)	98.48	98.48	98.10	100	98.80
Ramachandran allowed (%)	1.52	1.52	1.90	0.0	1.20
Ramachandran outliers (%)	0.0	0.0	0.0	0.0	0.0
Rotamer outliers (%)	2.52	0.71	0.71	0.64	0.0
Clashscore	1.17	2.84	1.15	4.12	0.36
Average *B* factor (Å^2^)					
Overall	24.57	22.49	27.93	16.35	15.73
Macromolecules	23.8	21.1	27.0	14.2	12.92
Ligands	48.8	16.6	22.5	22.8	21.8
Solvent	29.2	33.0	36.5	31.0	28.7
No. of TLS groups	16	20	—	—	—
PDB code	6vxs	6w02	6w6y	6wcf	6wen

† *R*_merge_ = 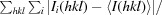 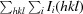, where *I_i_*(*hkl*) is the intensity of observation *i* of reflection *hkl*.

‡ As defined by Karplus & Diederichs (2012 ▸).

§ *R* = 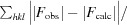 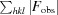 for all reflections, where *F*_obs_ and *F*_calc_ are observed and calculated structure factors, respectively. *R*_free_ is calculated analogously for the test reflections, which were randomly selected and excluded from the refinement.

¶ As defined by *MolProbity *(Chen *et al.*, 2010 ▸).

## References

[bb2] Atasheva, S., Akhrymuk, M., Frolova, E. I. & Frolov, I. (2012). *J. Virol.* **86**, 8147–8160. 10.1128/JVI.00733-12 PMC3421642 22623789

[bb3] Atasheva, S., Frolova, E. I. & Frolov, I. (2014). *J. Virol.* **88**, 2116–2130. 10.1128/JVI.03443-13 PMC3911523 24335297

[bb4] Báez-Santos, Y. M., St John, S. E. & Mesecar, A. D. (2015). *Antiviral Res.* **115**, 21–38. 10.1016/j.antiviral.2014.12.015 PMC5896749 25554382

[bb5] Bogoch, I. I., Watts, A., Thomas-Bachli, A., Huber, C., Kraemer, M. U. G. & Khan, K. (2020). *J. Travel Med.* **27**, taaa011. 10.1093/jtm/taaa011 PMC7074660 31985790

[bb6] Bredenbeek, P. J., Pachuk, C. J., Noten, A. F., Charité, J., Luytjes, W., Weiss, S. R. & Spaan, W. J. (1990). *Nucleic Acids Res.* **18**, 1825–1832. 10.1093/nar/18.7.1825 PMC330602 2159623

[bb13] Chen, V. B., Arendall, W. B., Headd, J. J., Keedy, D. A., Immormino, R. M., Kapral, G. J., Murray, L. W., Richardson, J. S. & Richardson, D. C. (2010). *Acta Cryst.* D**66**, 12–21. 10.1107/S0907444909042073 PMC2803126 20057044

[bb7] Cho, C.-C., Lin, M.-H., Chuang, C.-Y. & Hsu, C.-H. (2016). *J. Biol. Chem.* **291**, 4894–4902. 10.1074/jbc.M115.700542 PMC4777827 26740631

[bb8] Ciotti, M., Ciccozzi, M., Terrinoni, A., Jiang, W.-C., Wang, C.-B. & Bernardini, S. (2020). *Crit. Rev. Clin. Lab. Sci.*, https://doi.org/10.1080/10408363.2020.1783198. 10.1080/10408363.2020.1783198 32645276

[bb9] Claverie, J.-M. (2020). *Viruses*, **12**, 646.

[bb10] Coronaviridae Study Group of the International Committee on Taxonomy of Viruses (2020). *Nat. Microbiol.* **5**, 536–544.

[bb11] Crawford, K., Bonfiglio, J. J., Mikoč, A., Matic, I. & Ahel, I. (2018). *Crit. Rev. Biochem. Mol. Biol.* **53**, 64–82. 10.1080/10409238.2017.1394265 29098880

[bb12] Cui, J., Li, F. & Shi, Z.-L. (2019). *Nat. Rev. Microbiol.* **17**, 181–192. 10.1038/s41579-018-0118-9 PMC7097006 30531947

[bb15] Eckei, L., Krieg, S., Bütepage, M., Lehmann, A., Gross, A., Lippok, B., Grimm, A. R., Kümmerer, B. M., Rossetti, G., Lüscher, B. & Verheugd, P. (2017). *Sci. Rep.* **7**, 41746. 10.1038/srep41746 PMC5288732 28150709

[bb16] Egloff, M. P., Malet, H., Putics, A., Heinonen, M., Dutartre, H., Frangeul, A., Gruez, A., Campanacci, V., Cambillau, C., Ziebuhr, J., Ahola, T. & Canard, B. (2006). *J. Virol.* **80**, 8493–8502. 10.1128/JVI.00713-06 PMC1563857 16912299

[bb17] Emsley, P., Lohkamp, B., Scott, W. G. & Cowtan, K. (2010). *Acta Cryst.* D**66**, 486–501. 10.1107/S0907444910007493 PMC2852313 20383002

[bb18] Fontana, P., Bonfiglio, J. J., Palazzo, L., Bartlett, E., Matic, I. & Ahel, I. (2017). *eLife*, **6**, e28533. 10.7554/eLife.28533 PMC5552275 28650317

[bb19] French, S. & Wilson, K. (1978). *Acta Cryst.* A**34**, 517–525.

[bb20] Grunewald, M. E., Chen, Y., Kuny, C., Maejima, T., Lease, R., Ferraris, D., Aikawa, M., Sullivan, C. S., Perlman, S. & Fehr, A. R. (2019). *PLoS Pathog.* **15**, e1007756. 10.1371/journal.ppat.1007756 PMC6521996 31095648

[bb21] Hintze, B. J., Lewis, S. M., Richardson, J. S. & Richardson, D. C. (2016). *Proteins*, **84**, 1177–1189. 10.1002/prot.25039 PMC4983197 27018641

[bb22] Holm, L. & Rosenström, P. (2010). *Nucleic Acids Res.* **38**, W545–W549. 10.1093/nar/gkq366 PMC2896194 20457744

[bb23] Huynh, K. & Partch, C. L. (2015). *Curr. Protoc. Protein Sci.* **79**, 28.9.1–28.9.14. 10.1002/0471140864.ps2809s79 PMC4332540 25640896

[bb24] Jankevicius, G., Hassler, M., Golia, B., Rybin, V., Zacharias, M., Timinszky, G. & Ladurner, A. G. (2013). *Nat. Struct. Mol. Biol.* **20**, 508–514. 10.1038/nsmb.2523 PMC7097781 23474712

[bb25] Karplus, P. A. & Diederichs, K. (2012). *Science*, **336**, 1030–1033. 10.1126/science.1218231 PMC3457925 22628654

[bb26] Kelly, J. A., Olson, A. N., Neupane, K., Munshi, S., San Emeterio, J., Pollack, L., Woodside, M. T. & Dinman, J. D. (2020). *J. Biol. Chem.*, https://doi.org/10.1074/jbc.AC120.013449. 10.1074/jbc.AC120.013449 PMC7397099 32571880

[bb27] Koh, J., Shah, S. U., Chua, P. E. Y., Gui, H. & Pang, J. (2020). *Front. Med. (Lausanne)*, **7**, 295. 10.3389/fmed.2020.00295 PMC7300278 32596248

[bb28] Kuri, T., Eriksson, K. K., Putics, A., Züst, R., Snijder, E. J., Davidson, A. D., Siddell, S. G., Thiel, V., Ziebuhr, J. & Weber, F. (2011). *J. Gen. Virol.* **92**, 1899–1905. 10.1099/vir.0.031856-0 21525212

[bb29] Laskowski, R. A., MacArthur, M. W., Moss, D. S. & Thornton, J. M. (1993). *J. Appl. Cryst.* **26**, 283–291.

[bb30] Lee, H.-J., Shieh, C.-K., Gorbalenya, A. E., Koonin, E. V., La Monica, N., Tuler, J., Bagdzhadzhyan, A. & Lai, M. M. C. (1991). *Virology*, **180**, 567–582. 10.1016/0042-6822(91)90071-I PMC7131164 1846489

[bb31] Lei, J. & Hilgenfeld, R. (2016). *Virol. Sin.* **31**, 288–299. 10.1007/s12250-016-3742-4 PMC7090527 27245450

[bb32] Lei, J., Kusov, Y. & Hilgenfeld, R. (2018). *Antiviral Res.* **149**, 58–74. 10.1016/j.antiviral.2017.11.001 PMC7113668 29128390

[bb33] Li, C., Debing, Y., Jankevicius, G., Neyts, J., Ahel, I., Coutard, B. & Canard, B. (2016). *J. Virol.* **90**, 8478–8486. 10.1128/JVI.00705-16 PMC5021415 27440879

[bb1] Liebschner, D., Afonine, P. V., Baker, M. L., Bunkóczi, G., Chen, V. B., Croll, T. I., Hintze, B., Hung, L.-W., Jain, S., McCoy, A. J., Moriarty, N. W., Oeffner, R. D., Poon, B. K., Prisant, M. G., Read, R. J., Richardson, J. S., Richardson, D. C., Sammito, M. D., Sobolev, O. V., Stockwell, D. H., Terwilliger, T. C., Urzhumtsev, A. G., Videau, L. L., Williams, C. J. & Adams, P. D. (2019). *Acta Cryst.* D**75**, 861–877. 10.1107/S2059798319011471 PMC6778852 31588918

[bb34] Malet, H., Coutard, B., Jamal, S., Dutartre, H., Papageorgiou, N., Neuvonen, M., Ahola, T., Forrester, N., Gould, E. A., Lafitte, D., Ferron, F., Lescar, J., Gorbalenya, A. E., de Lamballerie, X. & Canard, B. (2009). *J. Virol.* **83**, 6534–6545. 10.1128/JVI.00189-09 PMC2698539 19386706

[bb35] Minor, W., Cymborowski, M., Otwinowski, Z. & Chruszcz, M. (2006). *Acta Cryst.* D**62**, 859–866. 10.1107/S0907444906019949 16855301

[bb36] Moss, J., Tsai, S.-C., Adamik, R., Chen, H.-C. & Stanley, S. J. (1988). *Biochemistry*, **27**, 5819–5823. 10.1021/bi00415a063 3179279

[bb37] Münnich, D., Békési, I. & Farkas, A. (1988). *Ther. Hung.* **36**, 109–114. 3249998

[bb38] Munnur, D., Bartlett, E., Mikolčević, P., Kirby, I. T., Rack, J. G. M., Mikoč, A., Cohen, M. S. & Ahel, I. (2019). *Nucleic Acids Res.* **47**, 5658–5669. 10.1093/nar/gkz305 PMC6582358 31216043

[bb39] Murshudov, G. N., Skubák, P., Lebedev, A. A., Pannu, N. S., Steiner, R. A., Nicholls, R. A., Winn, M. D., Long, F. & Vagin, A. A. (2011). *Acta Cryst.* D**67**, 355–367. 10.1107/S0907444911001314 PMC3069751 21460454

[bb40] Padilla, J. E. & Yeates, T. O. (2003). *Acta Cryst.* D**59**, 1124–1130. 10.1107/s0907444903007947 12832754

[bb41] Pehrson, J. R. & Fried, V. A. (1992). *Science*, **257**, 1398–1400. 10.1126/science.1529340 1529340

[bb42] Piotrowski, Y., Hansen, G., Boomaars-van der Zanden, A. L., Snijder, E. J., Gorbalenya, A. E. & Hilgenfeld, R. (2009). *Protein Sci.* **18**, 6–16. 10.1002/pro.15 PMC2708038 19177346

[bb43] Rack, J. G. M., Perina, D. & Ahel, I. (2016). *Annu. Rev. Biochem.* **85**, 431–454. 10.1146/annurev-biochem-060815-014935 26844395

[bb44] Rice, P., Longden, I. & Bleasby, A. (2000). *Trends Genet.* **16**, 276–277. 10.1016/s0168-9525(00)02024-2 10827456

[bb45] Saikatendu, K. S., Joseph, J. S., Subramanian, V., Clayton, T., Griffith, M., Moy, K., Velasquez, J., Neuman, B. W., Buchmeier, M. J., Stevens, R. C. & Kuhn, P. (2005). *Structure*, **13**, 1665–1675. 10.1016/j.str.2005.07.022 PMC7126892 16271890

[bb46] Snijder, E. J., Decroly, E. & Ziebuhr, J. (2016). *Adv. Virus Res.* **96**, 59–126. 10.1016/bs.aivir.2016.08.008 PMC7112286 27712628

[bb47] Tang, D., Comish, P. & Kang, R. (2020). *PLoS Pathog.* **16**, e1008536. 10.1371/journal.ppat.1008536 PMC7244094 32442210

[bb48] Thiel, V., Ivanov, K. A., Putics, A., Hertzig, T., Schelle, B., Bayer, S., Weissbrich, B., Snijder, E. J., Rabenau, H., Doerr, H. W., Gorbalenya, A. E. & Ziebuhr, J. (2003). *J. Gen. Virol.* **84**, 2305–2315. 10.1099/vir.0.19424-0 12917450

[bb49] Vagin, A. & Teplyakov, A. (2010). *Acta Cryst.* D**66**, 22–25. 10.1107/S0907444909042589 20057045

[bb50] Virdi, R. S., Bavisotto, R. V., Hopper, N. C. & Frick, D. N. (2020). *bioRxiv*, 2020.07.06.190413.

[bb51] Winn, M. D., Ballard, C. C., Cowtan, K. D., Dodson, E. J., Emsley, P., Evans, P. R., Keegan, R. M., Krissinel, E. B., Leslie, A. G. W., McCoy, A., McNicholas, S. J., Murshudov, G. N., Pannu, N. S., Potterton, E. A., Powell, H. R., Read, R. J., Vagin, A. & Wilson, K. S. (2011). *Acta Cryst.* D**67**, 235–242. 10.1107/S0907444910045749 PMC3069738 21460441

[bb52] Wojdyla, J. A., Manolaridis, I., Snijder, E. J., Gorbalenya, A. E., Coutard, B., Piotrowski, Y., Hilgenfeld, R. & Tucker, P. A. (2009). *Acta Cryst.* D**65**, 1292–1300. 10.1107/S0907444909040074 PMC7161637 19966415

[bb53] Wu, F., Zhao, S., Yu, B., Chen, Y.-M., Wang, W., Song, Z.-G., Hu, Y., Tao, Z.-W., Tian, J.-H., Pei, Y.-Y., Yuan, M.-L., Zhang, Y.-L., Dai, F.-H., Liu, Y., Wang, Q.-M., Zheng, J.-J., Xu, L., Holmes, E. C. & Zhang, Y.-Z. (2020). *Nature*, **579**, 265–269. 10.1038/s41586-020-2008-3 PMC7094943 32015508

[bb54] Xu, Y., Cong, L., Chen, C., Wei, L., Zhao, Q., Xu, X., Ma, Y., Bartlam, M. & Rao, Z. (2009). *J. Virol.* **83**, 1083–1092. 10.1128/JVI.01862-08 PMC2612350 18987156

