# Supplementary material for: Crystal structures of SARS-CoV-2 ADP-ribose phosphatase: from the apo form to ligand complexes
Source: IUCrJ. 2020 Jul 17;7(Pt 5):814–24. doi: 10.1107/S2052252520009653 (PMC7467174; doi:10.1107/S2052252520009653)
Supplement: Supplementary file 1 [file m-07-00814-sup1.pdf]

IUCrJ

Volume 7 (2020)

Supporting information for article:

Crystal structures of SARS-CoV-2 ADP-ribose phosphatase: from the apo form to ligand complexes

Karolina Michalska, Youngchang Kim, Robert Jedrzejczak, Natalia I. Maltseva, Lucy Stols, Michael Endres and Andrzej Joachimiak

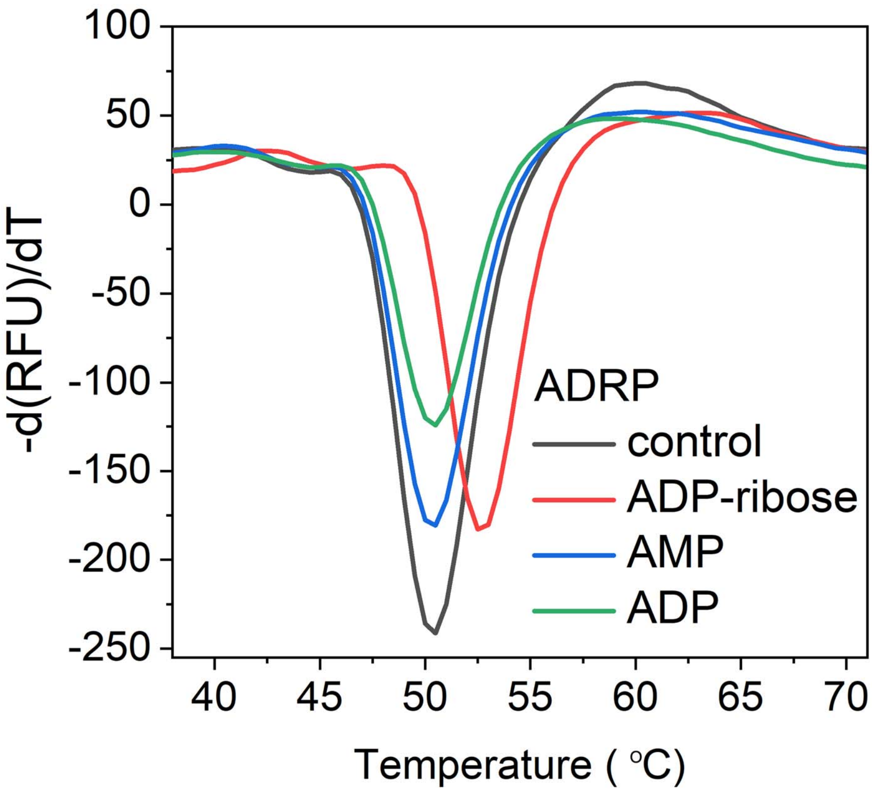

Figure S1 Thermal stability of ADRP protein probed using DSF in the presence AMP, ADP and ADP-ribose. ADRP samples were labeled with SYPRO orange dye (Huynh & Partch, 2015).
